# Phrenology

**Published:** 1860-07

**Authors:** J. W. Clift

**Affiliations:** Columbus, Ga.


### ARTICLE II.

*Phrenology.*—By J. W. CLIFT, Columbus, Ga.

Observing that an article on the above named subject from our pen, some months since, has elicited a reply from Dr. Taliaferro, in the Journal of April, a reply to him was intended last month, but owing to absence from the city, has been deferred until now.

When that article was penned by the writer, nothing was further from his mind than the supposition that he thereby merited the title of "Defender of Phrenology," and, here let it be understood, we are not yet radical enough to say *amen* to *every* idea which may be advanced in favor of Phrenology. We believe that, like all other systems of truth, or error, it will be greatly modified as time rolls on, in minor points, at least.

It would, indeed, be strange were it not so; nor do we know of any of its advocates who will claim to locate every organ "to the hundredth of an inch," or that every organ has yet been even discovered.

There are portions of the head about which the most noted Phrenologists claim to know but little. Even Fowler, radical as he is in his ideas on this, his favorite study, does not pretend to know *all* about Phrenology, or to cover all the head with diagrams. He says—"There are doubtless other organs yet undiscovered; Phrenology is yet in its infancy; though *perfect in itself*, our *knowledge* of it is *not* perfected; as every successive generation makes advances upon one another in Astronomy, Chemistry, and other departments of Science, so Gall and Spurzheim have only discovered the *landmarks* of this science, and have left much to be filled up by those who come after.

This, I believe, is, in the main, true; like all other things, Phrenology requires years of patient toil and study, ere it will *approximate perfection;* hence, to say that we can tell *exactly* in *every* instance, the disposition of *every* person we *meet*, would be the heighth of folly and egotism, and an error which Fowler—*Hydropath* though he be—has never committed, to my knowledge.

The general shape of the head, the locality of the several classes of organs and faculties, and, to use Fowler's expression, "the land-marks of the science"—the outline of Phrenology—has been given us, that the minds of future generations may modify and perfect. Were mind manifested independently from bodily or animal organization, by humanity at large, the case would be different. Physiology asserts her claim here, when man courts an investigation of the connection of mind with matter; and to say that by the scalpel we could prove to a mathematical certainty the truth of all which is claimed by the advocates of this system, few men would be found rash enough to assert; at least, the *writer* would as soon expect to be able to manufacture *thought* by *machine* as to take a scalpel, and, dividing the brain of a subject, find a portion directly beneath where Phrenologists locate Memory, and by dissection prove it to possess different characteristics, and to be composed of different material from a portion of brain taken from beneath Firmness. The relation existing between mind and matter, if we understand aught of it, is not demonstrable by the bare scalpel, whatever Gall and Spurzheim may have thought *years ago*. At that time, the anatomy and pathology of the nervous system was not so well understood as now; indeed, in no department of Medical Science have such strides onward been taken as here; in fact, the mass of the medical profession, thirty years ago, seem to have known little about nervous diseases, perhaps one reason being, mankind in civilized countries were not then the puny, delicate, nervous mortals who exist at present. Hence, Gall might then have thought anatomy would prove something for Phrenology; but we *insist* that the *proof* of the *truth* or *falsehood* of this "*theory*" rests not on anatomical investigation, but mainly in the fact that an entire stranger can look at another, examine him, and tell *almost to a certainty every single trait of character he possesses;* that this has been done in hundreds of instances, that it *can be done now*, that men are living now who can do it, and *are* doing it *every day*, *thousands will testify.*

Dr. T. must have been singularly unfortunate in his selection of a Phrenologist to examine his head, from his description of the performance.

The writer has had a different experience. O. S. Fowler examined his head in Savannah, in March, 1858, and it was done in haste, in his office, amid a crowd; he was a *perfect stranger* to Fowler and every person in the room, but one. With great rapidity he passed his hand over the head, dictating to an amanuensis, who took notes in short-hand on the chart. Had my own mother described the character of her boy it could not have been done more *correctly*, is admitted by all my friends who have seen the chart, and by the whole of my family. I have it now with me, and moreover would not sell the advice he gave at the time, pointing out the weak points in the character, &c., for the gold of Ophir. Although the writer has seen Mr. Fowler neither before nor since the time referred to, and for only about half an hour then, to speak with him, yet he feels greatly indebted to Phrenology, through him, for that chart.

This experience, so different from that related by Dr. T., *may* have prejudiced me in favor of Phrenology, at any rate, it has satisfied us, in common with others, who are willing to take such evidence, that the truth of Phrenology is sustained by an overwhelming mass of testimony of this kind, and we appeal to an enlightened and intelligent public whether this evidence is not indubitable proof of the truth of this system.

Again: If our opponent is unwilling to admit the difference of temperaments, and their modification of the faculties of the mind, as Phrenology—which doctrine has been one of the fundamental ones on which is based the whole system—he only leaves the bare *Crainology*, as it is termed, and desires us to prove from that alone the truth of a system, embracing man's organization, mentally, morally and physically; to use his own words, "refute physiological facts," or own our error, neither of which have we the remotest intention of doing, any more than we should have if he asked us to throw away all belief that there was a future state of existence for the soul after death, and then to prove the *existence of Heaven by showing it to him.*

Again: His answers to the questions propounded in my last article are very unsatisfactory. I asked, First. "If the brain continually acts as a unit, how and *why* can we exercise several faculties at one and the same time? He replies, "We *deny*" it, because, "else we might translate Euripides, solve one of Euclid's most difficult problems, and talk with our friends, *at one and the same time.*" Not so; he thus exercises the single faculty—*Memory*—on *three different subjects at the same time*, which no one ever claimed to do; certainly *we* did not in question first, but, "Why can we exercise several different faculties at the same instant" on *one subject?* was our meaning. He has not replied to it, in our opinion, at all, but to a totally different question.

In reply to question second—"Why is there such a thing known as a *Monomaniac?*"—he says, "*Monomania* cannot be established by pathological evidences in the brain." Why not, if the "Medico-Legal Review of the case of Patrick Maude," in the *Journal* of April, be correct? What was the evidence that *he* was a monomaniac? If the Scalpel did not *confirm* the evidence, before apparent, of his partial insanity, then we rely upon that evidence *entirely*, and what is

*more* to the point, *that very kind of evidence* CAN BE, and IS adduced in support of the system of *Phrenology*, and Dr. T. discards it, and calls the theory built upon *such* evidence "*speculative theory!!!*"

Again: Question third reads—"And why is Phrenology false, when it teaches that the intellectual faculties of the negro are so small that he is better fitted for manual labor than intellectual effort?" He replies, "We were not aware the negro's capacity was judged of by the *weight* or *bulk* of his head," &c. I leave it with the candid reader to judge whether the obvious meaning of the question was not, Why are *negroes*, *as a race*, possessed of very little forehead, in proportion to the head behind a perpendicular line drawn through the center of the ear? Phrenology, in one of its first principles, teaches that, as a *general rule*, the head before this line —viz., forehead and top head, or Moral and Intellectual organs—are proportioned to the faculties corresponding with them there, and *behind* that line, a like rule applied, proportions the *Animal faculties*. *We* say, applying that principle of Phrenology to the African head, is a proof of its truth or falsehood; and the other principles of our "*theory*" will be found to apply as well. So much for his reply to *that* question.

We now leave the subject under discussion, at present, with the remark, that we were much pleased with the promptness of our opponent in defending his opinions. He wields an able pen, and long may he live to defend the truths of Science, though we thereby run a risk of again encountering some of his sarcastic shafts aimed at our devoted head, if we unfortunately differ with him.

